# Ethylene glycol intoxication following brake fluid ingestion complicated with unilateral facial nerve palsy: a case report

**DOI:** 10.1186/s13256-019-2139-z

**Published:** 2019-07-03

**Authors:** B. M. D. B. Basnayake, A. W. M. Wazil, N. Nanayakkara, R. M. B. S. S. Mahanama, P. N. S. Premathilake, K. K. M. C. D. K. Galkaduwa

**Affiliations:** 1 Department of Nephrology and Renal Transplant, Teaching Hospital Kandy, Kandy, Sri Lanka; 2 Department of Medicine, Teaching Hospital Kandy, Kandy, Sri Lanka

**Keywords:** Ethylene glycol, Brake fluid, Intoxication, Facial nerve palsy

## Abstract

**Background:**

Brake oil is an automobile transmission fluid composed of a mixture of toxic alcohols such as ethylene glycols and glycol ethers. Both accidental and intentional ingestion cases have been reported and they can present with multisystem involvement. Life-threatening complications evolve from deleterious effects on cardiopulmonary and renal systems. Effects on neurological and gastrointestinal systems give rise to a multitude of complications although non-fatal in nature. The biochemical panel consists of a high concentration of ethylene glycol with severe metabolic acidosis, high anion gap, high osmolar gap, oxaluria, and hypocalcemia. The mainstay of treatment is enhanced elimination of ethylene glycol and its metabolites by hemodialysis, together with general supportive care, gastric decontamination, and vitamins such as thiamine and pyridoxine to minimize the adverse effects of intoxication.

**Case presentation:**

A 26-year-old Sinhalese woman presented with reduced urine output, shortness of breath, reduced level of consciousness, abdominal pain, and vomiting with mild degree fever of 2 days’ duration. She had bilateral lower limb edema, crepitations over bilateral lower lung fields, and right-sided lower motor type facial nerve palsy. Investigations showed severe metabolic acidosis with high anion gap and high osmolar gap. With regular hemodialysis she made a complete recovery after 3 months.

**Conclusion:**

Even without a clear history of poisoning, the presence of a high anion, high osmolar gap metabolic acidosis should prompt one to search for ethylene glycol ingestion. Uncommon manifestations like cranial neuropathies need to be examined and considered. Timely aggressive treatment leads to a better prognosis.

## Background

Brake fluid is a type of transmission oil used in hydraulic brake and hydraulic clutch applications in automobiles. It is composed of a mixture of toxic alcohols such as ethylene glycols and glycol ethers. Ethylene glycol is also used in antifreeze, coolants, and chemical solvents. Ethylene glycol intoxication is not a rare event and both accidental and intentional poisoning have been reported [1, 2]. However, most deaths occur from intentional poisoning and deaths due to accidental ingestion in children are extremely rare [3]. Toxic manifestations are caused by the metabolites of ethylene glycol rather than the agents themselves, resulting in a gap between the time of ingestion and the onset of clinical features of toxicity [4]. Intoxicated patients will have neurological, gastrointestinal, cardiopulmonary, and renal manifestations during the course [2]. We report a case of ethylene glycol intoxication following brake fluid ingestion complicated with acute renal failure, unilateral facial nerve palsy, gastrointestinal manifestations, and cardiopulmonary manifestations.

## Case presentation

A 26-year-old Sinhalese woman was transferred from a local hospital with a history of reduced urine output, shortness of breath, reduced level of consciousness, abdominal pain, vomiting, and mild degree fever of 2 days’ duration. Her bilateral lower limbs were edematous but she was not pale or icteric. Her pulse rate was 112 beats per minute and blood pressure was 140/70 mmHg. An abdominal examination did not reveal organomegaly. Bilateral lower zone crepitations were noted on lung auscultation. Her respiratory rate was 20 cycles per minute and oxygen saturation was 97% on air. She had right-sided lower motor type facial nerve palsy. Glasgow Coma Scale was 13/15. The rest of the neurological examination including other cranial nerves and ophthalmoscope examination was unremarkable.

Our initial working diagnosis was leptospirosis with acute kidney injury and treatment was initiated accordingly (intravenously administered antibiotic and hemodialysis via femoral vascular catheter), but we could not explain the cranial nerve involvement. The following day she came out with the history of a suicide attempt in which she had self-ingested brake oil (amount not clear) after a conflict with her husband.

On admission her renal functions were deranged with serum creatinine of 352 μmol/ L, blood urea of 14.1 mmol/l, Na+ 140 mmol/l, and K 5.2 mmol/l. Arterial blood gas showed pH 7.08, partial pressure of oxygen (PO_2_) 94, partial pressure of carbon dioxide (PCO_2_) 28, bicarbonate (HCO_3_) 13.8, and base excess − 18 mEq per liter. Her serum osmolality was 339 mosmols with an osmolar gap of 20 mOsm/kg and anion gap was 32 mEq/l. Although relevant, her urine was not examined for calcium oxalate crystals. Full blood count showed hemoglobin of 12.7 g/dl, platelet of 185 × 10^6^/L, and white cell count of 15.2 × 10^6^/L. Her C-reactive protein was 22 mg/dl. Her random blood sugar was 92 mg/dl. Her blood and urine cultures were negative. Her pro-calcitonin levels were within normal range. A chest X-ray did not reveal any abnormality such as consolidation or pleural effusion. A non-contrast computed tomography (CT) scan of her brain was normal. *Leptospira* antibody tested after 10 days of disease was negative. She was started on initial consecutive daily dialysis followed by every other day dialysis which yielded a considerable improvement in renal functions. After 10 days of hospital stay she was discharged with residual facial nerve palsy. Over the course of 3 months’ clinic follow-up she had complete renal and neurological improvement.

## Discussion

Ethylene glycol is water soluble and toxic. Ethylene glycol is excreted by the kidneys; it has a half-life of 7 to 10 hours. However, its metabolites, such as glycolaldehyde, glycolic acid, and oxalic acid, have longer half-lives and remain in the body for several days and their highly toxic effects result in toxic clinical features [5–7]. Individuals who ingest ethylene glycol may present to an emergency department with complaints of confusion, difficulty in walking (ataxia), hallucinations, and slurred speech. They may also have gastrointestinal symptoms like nausea, vomiting, and abdominal pain. Sometimes the presentation may be tetany and seizures. Renal involvement may develop within 24 to 72 hours. If left untreated it is usually fatal within 24 to 36 hours [7, 8]. Ethylene glycol toxicity is divided into three distinct stages; however, not all individuals will develop all three phases in the relevant timeframe. The phases include central nervous system (CNS) involvement (0.5–12 hours), cardiopulmonary toxicity (12–36 hours), and renal toxicity phase (24–72 hours) [2].

Neurological manifestations include slurred speech, ataxia, nystagmus, dysarthria, dysphagia, somnolence, visual disturbances, areflexia, myoclonic jerks, cerebral edema, and seizures. At high doses it can cause CNS depression leading to coma and brain death [2, 9–11]. There are reported cases with multiple cranial nerves involvement following ethylene glycol intoxication, such as bilateral facial nerve palsies and dysfunction of cranial nerves ΙΙ, Ѵ, ѴΙΙΙ, ΙΧ, Χ, and ΧΙΙ [11–13]. Neurological effects occurring early in the course are due to direct action of ethylene glycol but, with time, accumulation of toxic metabolites contributes to other manifestations. Encephalopathy and cerebral edema will lead to persistent coma whereas seizures may be due to direct CNS toxic effects or hypocalcemia [2, 14, 15]. The etiology of cranial nerve palsies is not fully understood, but suggested mechanisms postulate oxalate crystal deposition or ethylene glycol-related pyridoxine dysfunction as the culprit [13].

Cardiopulmonary features include tachycardia, high blood pressure, tachypnea, congestive cardiac failure, and prolongation of QT interval in electrocardiography (ECG). Fatality is high in this stage. These effects are mediated by concentrated metabolites of ethylene glycol and hypocalcemia [9, 16]. Postmortem findings in fatal cases have demonstrated calcium oxalate crystals in myocardial tissue and focal hemorrhages [17].

Renal involvement is characterized by acute kidney injury comprising hematuria, proteinuria, renal tubular necrosis, loin pain, reduced urine output, and anuria. Patients require hemodialysis initially. The insult is usually reversible within weeks but might, rarely, take months [2, 18]. Other rare manifestations include leukocytosis or bone marrow suppression resulting in pancytopenia [18].

A definitive diagnosis can be made by measuring the serum concentration of ethylene glycol, but it is cumbersome and time consuming. In a patient with a history or suspicion of ingesting ethylene glycol an arterial blood gas analysis with a pH less than 7.3, a serum bicarbonate level less than 20 mmol/L, osmolar gap of more than 10 mOsm/L, and the presence of urinary oxalate crystals are highly suggestive of intoxication [15]. Our patient had all the features except the presence of urine oxalate crystals as it was not performed at the time. Osmolar gap is due to the presence of ethylene glycol itself and not due to its metabolites. Therefore, the osmolar gap will gradually decline while the metabolite concentration and toxic effects rise. Patients may also develop hypocalcemia as the concentration of oxalate increases [2, 4].

Treatments of ethylene glycol poisoning include stabilization, gastric decontamination, vitamins to minimize complications, inhibition of metabolism, and enhanced elimination [2, 19]. The standard stabilization includes securing the airway, breathing, and circulation. Some physicians practice corrections of severe metabolic acidosis, at least in part, with sodium bicarbonate but the cautions are hypocalcemia and hypernatremia. Seizures need therapy with benzodiazepines [2, 20].

Gastric decontamination is not universally recommended as ethylene glycol absorption is fast and patients are drowsy when presenting to hospital carrying a high risk of aspiration. However, if a patient presents early following ingestion, it should theoretically be acceptable to proceed with lavage to remove unabsorbed ethylene glycol [20].

Ethylene glycol is metabolized by alcohol dehydrogenase. Inhibition of the enzymatic action can minimize the metabolism and formation of toxic metabolites. Fomepizole is highly effective in inhibiting alcohol dehydrogenase. Ethanol is a competitor for alcohol dehydrogenase with a greater affinity rendering it useful to inhibit ethylene glycol metabolism [15]. Vitamins like thiamine and pyridoxine prevent the formation of oxalic acid by facilitating the conversion of glyoxylic acid (toxic metabolite of ethylene glycol) to non-toxic metabolites [2, 15].

The mainstay of management is the elimination of non-metabolized ethylene glycol and metabolites from blood by hemodialysis. This will additionally correct the other metabolic derangements caused by ethylene glycol poisoning. It is recommended traditionally to offer hemodialysis if the ethylene glycol level is more than 500 mg/L. Furthermore, hemodialysis is indicated if the patient has severe metabolic acidosis, renal failure, severe electrolyte disturbances, or a generally deteriorating condition despite supportive measures with any serum ethylene glycol levels. The recommendation is to continue hemodialysis until the concentration drops below 500 mg/L [15, 20–23]. Due to unavailability of resources we could not monitor the ethylene glycol levels with hemodialysis. Therefore, therapy was mainly guided by clinical status, renal functions with electrolytes and metabolic acidosis, and hemodialysis was continued until the monitored parameters were improved.

## Conclusion

Ethylene glycol intoxication is a potential life-threatening condition and if the patient reveals history of ingestion, an immediate and aggressive protocol-based management needs to be instituted. However, in the usual scenario when the patient denies self-poisoning by ingestion, it is crucial to interpret the available investigations of severe metabolic acidosis, high anion gap, high osmolar gap, and oxaluria in order to arrive at the accurate etiology. Unusual presentations like cranial neuropathies need to be checked out and considered.

## Abbreviations

CNS: Central nervous system
CT: Computed tomography
ECG: Electrocardiography
HCO_3_: Bicarbonate
PCO_2_: Partial pressure of carbon dioxide
PO_2_: Partial pressure of oxygen
